# Kinematic alignment, but not mechanical alignment, preserves the knee–ankle relationship after total knee arthroplasty: A retrospective radiographic analysis from the FP‐UCBM Knee Study Group

**DOI:** 10.1002/ksa.70138

**Published:** 2025-10-28

**Authors:** Edoardo Franceschetti, Giancarlo Giurazza, Marco Donantoni, Stefano Campi, Andrea Tanzilli, Pietro Gregori, Michele Paciotti, Biagio Zampogna, Andrea Marinozzi, Umile Giuseppe Longo, Rocco Papalia

**Affiliations:** ^1^ Fondazione Policlinico Universitario Campus Bio‐Medico Roma Italy; ^2^ Department of Medicine and Surgery Research Unit of Orthopaedic and Trauma Surgery, Università Campus Bio‐Medico di Roma Roma Italy

**Keywords:** coronal ankle alignment, kinematic alignment, knee–ankle relationship, mechanical alignment, total knee arthroplasty

## Abstract

**Purpose:**

Several alignment strategies have been proposed in total knee arthroplasty (TKA), with the two extremes being mechanical alignment (MA) and unrestricted kinematic alignment (KA). While MA standardises coronal knee parameters to achieve a neutral alignment, KA reproduces each patient's native joint lines. The impact of these strategies on the ankle joint remains poorly understood. The aim of this study was to compare the effects of MA and KA on coronal ankle alignment, hypothesising that MA induces greater alterations than KA.

**Methods:**

In this retrospective cohort study, 208 consecutive TKA cases, performed with either MA (*n* = 104) or unrestricted KA (*n* = 104) were included. Preoperative and 1‐year postoperative full‐length standing radiographs were analysed for lateral distal femoral angle (LDFA), medial proximal tibial angle (MPTA), hip–knee–ankle angle (HKA), Coronal‐Plane‐Alignment‐of‐the‐Knee (CPAK) categories and ankle‐specific parameters: talar inclination (TI), tibial plafond inclination (TPI), and talar tilt (TT). Changes in radiographic parameters (Δ) were calculated as the difference between 1‐year postoperative and preoperative values. Within‐group and between‐group changes were assessed with paired and independent t‐tests, respectively, and correlations between knee and ankle changes were calculated. Statistical significance was set at *p* < 0.05.

**Results:**

MA resulted in significant postoperative changes in TI (–3.21 ± 3.28°; *p* < 0.001) and TPI (–2.62 ± 3.16°; *p* < 0.001), whereas KA produced no significant changes (TI: –0.60 ± 3.54°, TPI: –0.18 ± 3.23°; *p* > 0.05). No significant changes were observed for TT in either group. In the MA group, ΔTPI correlated moderately with ΔHKA, ΔLDFA, and ΔMPTA. Pre/postoperative changes in CPAK category did not show any impact on coronal ankle parameters following TKA (*p* > 0.05)

**Conclusions:**

Coronal ankle alignment is preserved by unrestricted KA but altered by MA. These findings suggest that KA minimises unintended ankle alterations, maintaining the patient‐specific knee–ankle relationship and potentially reducing the risk of secondary ankle malalignment or degeneration following TKA.

**Level of Evidence:**

Level IV retrospective analysis.

**Abbreviations**
AOA: ankle osteoarthritis
FA: functional alignment
HKA: hip–knee–ankle angle
KA: unrestricted kinematic alignment
LDFA: lateral distal femoral angle
MA: mechanical alignment
MPTA: medial proximal tibial angle
PACS: picture‐archiving communication system
TI: talar inclination
TKA: total knee arthroplasty
TPI: tibial plafond inclination
TT: talar tilt

## INTRODUCTION

Several alignment strategies have been proposed in total knee arthroplasty (TKA), with the two extremes being mechanical alignment (MA) [22] and unrestricted kinematic alignment (KA) [14]. MA positions the femoral and tibial components perpendicular to their respective mechanical axes, aiming to obtain a neutral hip–knee–ankle (HKA) angle of 180°. In contrast, KA—introduced by Howell in the early 2000s in response to the relatively high dissatisfaction rate observed with MA [1, 12]—aims to reproduce the patient's pre‐arthritic joint lines and native limb alignment by respecting the natural orientation of the articular surfaces of femur and tibia.

Therefore, while MA standardises coronal knee parameters—represented by the medial proximal tibial angle (MPTA), lateral distal femoral angle (LDFA), and overall HKA angle— across patients, KA preserves each patient's natural joint line obliquity and ligament balance.

By variably altering the coronal orientation of the tibia and femur, TKA alignment strategies may affect not only knee biomechanics but also adjacent joints, particularly the ankle [20]. Changes in tibial orientation can in fact modify ankle joint loading and potentially induce secondary malalignment or symptoms [17, 25]. However, knowledge of these downstream effects remains limited and, although the effects of MA on the knee–ankle relationship have been partially investigated, the impact of unrestricted KA remains largely unexplored [18, 21].

The aim of this study was therefore to compare the effects of mechanical and unrestricted kinematic alignment on coronal ankle alignment, with the hypothesis that MA induces greater alterations than KA.

## MATERIALS AND METHODS

### Study design and participants

Institutional review board approval (IRB No. 32.19 OSS) was granted for this retrospective cohort study, which was conducted in accordance with the Declaration of Helsinki. Written consent was obtained from all included participants. Patients with Kellgren–Lawrence grade IV knee osteoarthritis undergoing TKA at our Institution (Fondazione Policlinico Universitario Campus Bio‐Medico) with either mechanical or unrestricted kinematic alignment technique and a minimum 1‐year follow‐up, were deemed eligible for inclusion. Exclusion criteria included a history of major surgery on the affected limb (e.g., osteotomies or fractures), absence or poor quality of full‐length anteroposterior preoperative and 1‐year postoperative radiographs, neurological conditions affecting gait, or evidence of severe ankle deformities. The study group consisted of 100 consecutive patients (104 knees) who underwent unrestricted calipered KA TKA [2, 6, 8, 9] between December 2022 and June 2023. The control group consisted of 98 consecutive patients (104 knees) who underwent manual MA TKA [5] between November 2021 and May 2022. The same implant design (GMK Sphere, Medacta) with PCL preservation was used in both groups, and all surgeries were performed by the senior authors, experienced in both techniques. None of the patients had other lower limb surgeries between the TKA and the final follow‐up visit.

### Radiographic analysis

Preoperative and 1‐year postoperative standing full‐length radiographs [10] were obtained following a standardised acquisition protocol. To ensure accurate hindfoot and ankle alignment measurements, long‐leg radiographs were acquired with patients standing barefoot in a standardised position with feet pointing forward, knees fully extended, and weight evenly distributed. Positioning was supervised by trained radiology staff to ensure consistency across all acquisitions.

Knee radiographic analysis included the following measurements: lateral distal femoral angle (LDFA), medial proximal tibial angle (MPTA), and hip‐knee‐ankle angle (HKA). MPTA and LDFA were used to calculate the Joint Line Obliquity (JLO) and the arithmetic hip‐knee‐ankle angle (aHKA) and patients were classified into coronal plane alignment of the knee (CPAK) categories accordingly.

**Figure 1:**
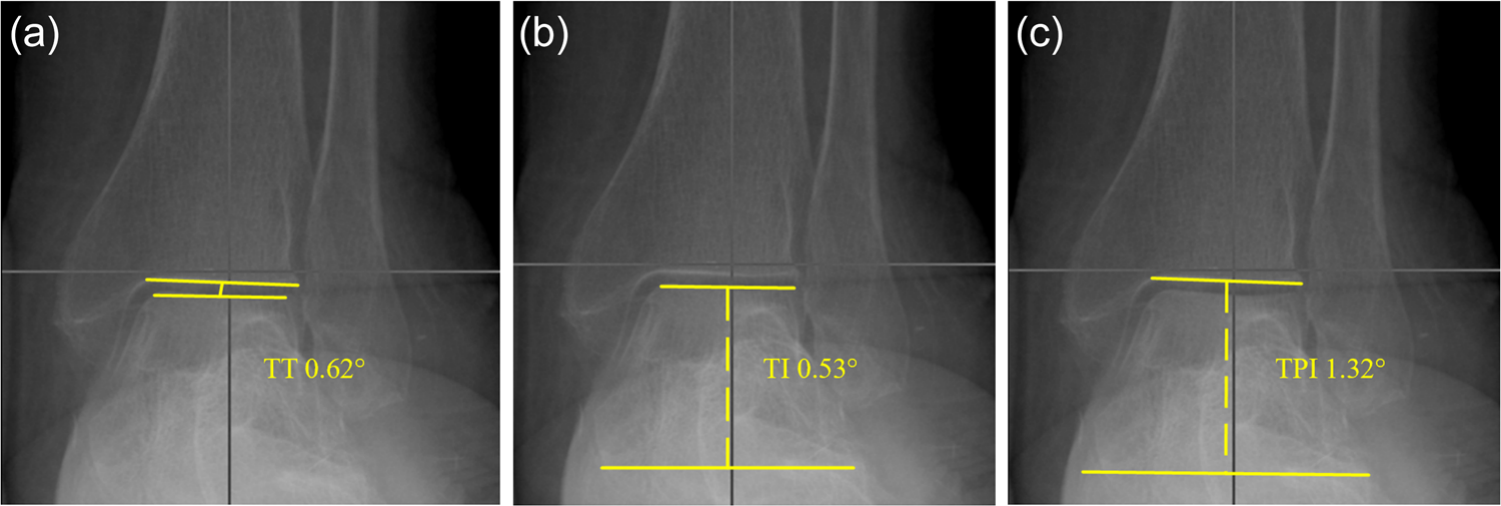
Ankle measurements on standing radiographs: (a) talar tilt (TT), (b) talar inclination angle (TI), and (c) tibial plafond inclination angle (TPI).

Ankle radiographic analysis (Figure 1) included three parameters: talar inclination (TI), defined as the angle between the horizontal plane and the superior surface of the talus; tibial plafond inclination (TPI), defined as the angle of the tibial plafond relative to the horizontal plane; and talar tilt (TT), defined as the angle between the tibial plafond and the talar dome. Varus and valgus ankle alignment were indicated by positive and negative angular values, respectively, with deformities considered severe if they exceeded 4° or were less than −4°.

All measurements were conducted by a single observer (M.D.) using the tools available in a picture‐archiving communication system (PACS) and recorded to the nearest 0.1°. Two separate measurements were taken six weeks apart, with an ICC of 0.87. Additionally, a second researcher (Ax.M.) repeated the measurements on 30 patients to determine inter‐observer reliability (ICC of 0.84).

### Data analyses

Descriptive statistics were performed to describe mean and standard deviation (SD) for all variables. All data analyses were performed using STATA 18 Software (StataCorp LLC, Lakeway Drive College Station, Texas, USA). Changes in radiographic parameters (Δ) were calculated as the difference between 1‐year postoperative and preoperative values. Within‐group changes were assessed using paired *t*‐tests, while between‐group differences were evaluated using independent *t*‐tests. Correlations between ΔTT, ΔTI, and ΔTPI and ΔLDFA, ΔMPTA, and ΔHKA were performed using Pearson's coefficients. Statistical significance was set at *p* < 0.05.

Post hoc power analyses for ΔTI and ΔTPI between the KA and MA groups were performed and found to be greater than 95%, indicating that the sample size was more than sufficient to detect clinically relevant differences between the two alignment strategies.

## RESULTS

**Figure 2:**
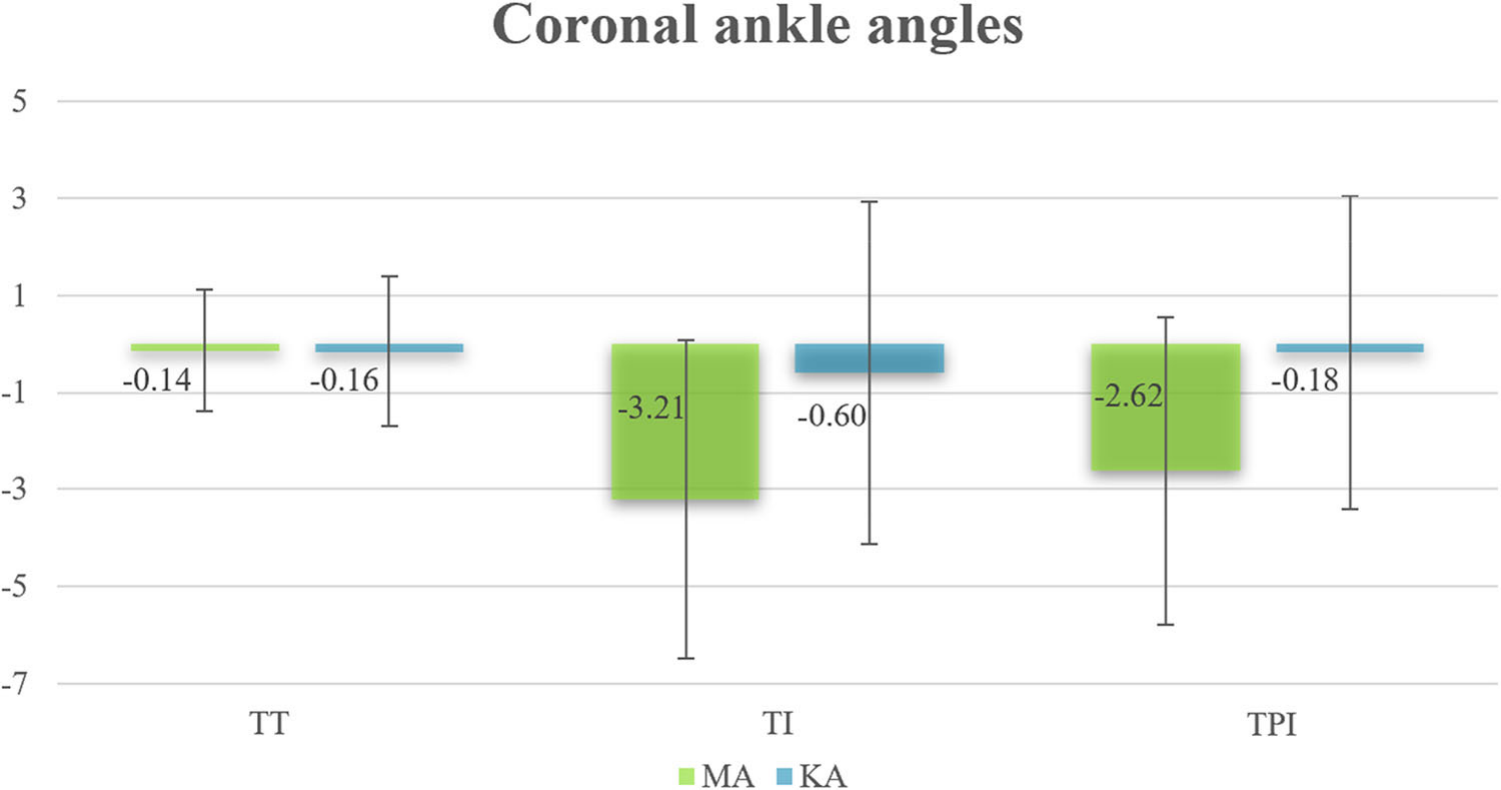
Change (postoperative − preoperative) in coronal ankle angles after kinematically (KA) and mechanically (MA) aligned total knee arthroplasty. TI, talar inclination; TPI, tibial plafond inclination; TT, talar tilt.

Mean changes (postoperative − preoperative) in TI and TPI differed significantly between alignment strategies. In the KA group, no significant changes were observed for TI (–0.60  ±  3.54°; *p* = 0.087) and TPI (−0.18  ±  3.23°; *p* = 0.531). In contrast, the MA group showed significant changes in TI (−3.21  ±  3.28°; *p* < 0.001) and TPI (–2.62  ±  3.16°; *p* < 0.001). In none of the groups did the TT change significantly (KA: −0.16  ±  1.55°, *p* = 0.253; MA: −0.14  ±  1.25°; *p* = 0.213) (Table 1, Figure 2). ΔTI and ΔTPI differed significantly between the MA and KA groups (*p* < 0.001), whereas ΔTT did not (*p* = 0.913) (Table 2). Post‐operative LDFA and MPTA differed significantly between the MA and KA group (*p* < 0.001) (Table 3). Only in the MA group the ΔTPI showed moderate, significant correlations with coronal knee parameters: ΔHKA (*p* = 0.006), ΔLDFA (*p* = 0.012), and ΔMPTA (*p* = 0.026) (Table 4). No significant differences in ΔTT, ΔTI or ΔTPI were observed between patients who shifted to a different CPAK category after TKA and those who remained in the same category (*p* > 0.05) (Table 5).

**Table 1:** Change (postoperative − preoperative) in coronal ankle angles in the mechanically aligned (MA) and kinematically aligned (KA) groups.

Parameter	Alignment	Mean Δ±SD (°)	*p*‐Value
TT	MA	−0.14 ± 1.25	0.262
	KA	−0.16 ± 1.55	0.302
TI	MA	−3.21 ± 3.28	<0.001
	KA	−0.60 ± 3.54	0.087
TPI	MA	−2.62 ± 3.16	<0.001
	KA	−0.18 ± 3.23	0.574

Abbreviations: TI, talar inclination; TPI, tibial plafond inclination; TT, talar tilt.

**Table 2:** Comparison of changes (postoperative − preoperative) in coronal ankle angles between the mechanically aligned (MA) and kinematically aligned (KA) groups.

Parameter	Mean Δ±SD (MA)	Mean Δ±SD (KA)	*p*‐Value
TT	−0.14 ± 1.25	−0.16 ± 1.55	0.913
TI	−3.21 ± 3.28	−0.60 ± 3.54	<0.001
TPI	−2.62 ± 3.16	−0.18 ± 3.23	<0.001

Abbreviations: TI, talar inclination; TPI, tibial plafond inclination; TT, talar tilt.

**Table 3:** Preoperative and postoperative knee coronal plane values in the mechanically aligned (MA) and kinematically aligned (KA) groups.

Parameter	Mean ± SD (MA)	Mean ± SD (KA)	*p*‐Value
Pre LDFA	88.61 ± 3.92	88.82 ± 3.36	0.679
Pre MPTA	88.12 ± 4.42	87.43 ± 3.81	0.231
Pre HKA	177.43 ± 9.24	178.01 ± 9.03	0.652
Post LFDA	90.22 ± 1.21	88.85 ± 3.58	<0.001
Post MPTA	89.73 ± 1.12	87.77 ± 2.57	<0.001
Post HKA	179.51 ± 1.01	179.05 ± 3.33	0.182

Abbreviations: HKA, hip‐knee‐ankle angle; LDFA, lateral distal femoral angle; MPTA, medial proximal tibial angle.

**Table 4:** Correlation between changes (postoperative – preoperative) in coronal ankle and coronal knee parameters between mechanical alignment and kinematic alignment.

Ankle angle	Knee parameter	Mechanical alignment		Kinematic alignment	
		Pearson *r*	*p*‐Value	Pearson *r*	*p*‐Value
ΔTT	ΔLDFA	0.220	0.167	−0.048	0.727
	ΔMPTA	0.045	0.778	−0.113	0.401
	ΔHKA	−0.114	0.479	−0.038	0.784
ΔTI	ΔLDFA	0.085	0.592	−0.180	0.179
	ΔMPTA	0.313	0.052	−0.048	0.724
	ΔHKA	0.231	0.147	−0.065	0.609
ΔTPI	ΔLDFA	−0.387	0.012	−0.057	0.659
	ΔMPTA	0.348	0.026	−0.092	0.457
	ΔHKA	0.421	0.006	−0.056	0.669

Abbreviations: HKA, hip‐knee‐ankle angle; LDFA, lateral distal femoral angle; MPTA, medial proximal tibial angle; TI, talar inclination; TPI, tibial plafond inclination; TT, talar tilt.

**Table 5:** Ankle parameters changes in patients with and without postoperative CPAK modification.

Parameter	Modified CPAK	Unmodified CPAK	*p*‐Value
ΔTT	−0.22 ± 1.41	−0.10 ± 1.32	0.531
ΔTI	−2.41 ± 3.42	−1.62 ± 3.31	0.112
ΔTPI	−1.92 ± 3.32	−1.13 ± 3.22	0.104

Abbreviations: TI, talar inclination; TPI, tibial plafond inclination; TT, talar tilt.

## DISCUSSION

The main result of the current study was that coronal ankle alignment—represented by the talar inclination and the tibial plafond inclination—is preserved by unrestricted KA TKA, but not by MA TKA.

Our findings reinforce the concept that TKA alignment strategy influences load distribution across the entire kinetic chain, affecting ankle joint orientation and loading: [7] the significant correlations observed in the MA group between ankle changes (ΔTPI) and coronal knee parameters (ΔHKA, ΔLDFA and ΔMPTA) suggest a biomechanical link between knee correction and ankle orientation and supports the notion that restoring native knee alignment limits perturbation of the distal joint.

Noteworthy, no significant differences in ΔTT, ΔTI or ΔTPI were found between patients who shifted to a different CPAK category after TKA and those who remained in the same category. This aligns with the findings of Sangaletti et al. [24] and underscores the inherent limitations of the CPAK classification, being a qualitative rather than quantitative system. As a result, patients classified within the same CPAK category may still present markedly different LDFA or MPTA values, meaning that more subtle correlations involving LDFA and MPTA can be masked by this classification.

Previous literature has shown that correction of varus or valgus deformity through MA can indeed improve certain ankle parameters, such as talar tilt, but often at the cost of creating new incongruencies [7, 15]. Jin et al. [15], in evaluating the effects of changes in LDFA and MPTA on ankle alignment, reported that varus corrections greater than 10° aggravated ankle varus incongruence. Gursu et al. [13] reported that acute correction of long‐standing severe varus deformities with MA TKA may lead to radiographic problems at the ankle joint, frequently associated with new onset pain after surgery, particularly when preoperative deformity exceeded 15°, suggesting that the ankle joint's ability to accommodate acute changes induced by MA is both variable and limited, and may be further reduced when hindfoot deformities become stiff over time [3]. Noteworthy, approximately 30% of patients with knee osteoarthritis undergoing TKA also present with concurrent ankle osteoarthritis (AOA) [3, 16]. In these patients, MA TKA has been shown to significantly affect gait parameters, as demonstrated by Kikuchi et al. [16]. Similarly, Chang et al. [3] reported that patients with concurrent AOA tend to experience worsening ankle and hindfoot symptoms after MA TKA. Moreover, AOA is often associated with reduced joint flexibility due to joint space narrowing and osteophyte formation, which may decrease the ability to adapt to altered load distribution following TKA [26, 27]. Graef et al. [11] further demonstrated that, among patients undergoing MA TKA, limited subtalar joint range of motion was associated with postoperative ankle pain progression. Choudhury et al. [4] demonstrated that mechanically aligned TKA for severe varus deformity significantly increases medial talar tilt, producing lateral tibio‐talar incongruency and medial overload.Additionally, Lee et al [19] and Rühling et al. [23] reported new or progressive ankle pain and new or progressive ankle arthritis after TKA in 23% and 21.8% of patients respectively, with higher rates in the varus group, particularly when preoperative talar tilt was present and large correction angles were applied.

Mechanical alignment imposes a 'one‐size‐fits‐all' neutral position, which in constitutional non‐neutral patients may generate pathological loading at the ankle and increase the risk of ankle arthritis progression [15, 19]. Against this background, the concept of restoring constitutional alignment through KA offers a biomechanically consistent rationale. Kim et al. [18] showed that postoperative ankle joint line orientation after KA TKA more closely resembles that of healthy controls than after MA TKA, with the tibial plafond and talar dome remaining nearly horizontal to the ground. Similarly, in a recent study comparing functional alignment (FA) with adjusted mechanical alignment, Sangaletti et al. [24] showed that FA was associated with smaller changes in TI, TPI and TT. Additionally, Onoi et al. [21] reported that, compared with MA, KA resulted in a more neutral ground mechanical axis during weightbearing, a more balanced distribution of coronal hindfoot pressure during walking, and greater stability in unipedal stance. Taken together, this data supports the notion that personalised strategies aiming to accommodate each patient's native knee alignment can mitigate the impact of TKA on the ankle, preserve ankle and hindfoot orientation and congruency, potentially lowering the risk of secondary ankle degeneration.

The strength of the present study lies in its direct comparison of two techniques—MA and unrestricted KA—that represent the opposite ends of the spectrum of alignment strategies in TKA. Importantly, no selection criteria were applied based on the degree of preoperative deformity, which allowed us to more clearly highlight differences that might have been obscured if “intermediate” approaches such as restricted KA, or adjusted MA had been included. On the other hand, some limitations should be acknowledged. It is a single‐centre, retrospective cohort study in which the control group underwent surgery before the study group. However, data collection was consistent, and both groups included comparable populations of knee osteoarthritic patients undergoing surgery by a limited number of experienced operators and following the same postoperative protocol and standard of care. We therefore believe that both temporal bias and learning curve‐related bias were adequately minimised.

Additionally, our analysis was restricted to radiographic parameters. Although ankle‐specific clinical scores are available, they were not collected in this study, as patients were not originally enroled with the intention of assessing ankle outcomes. Future studies incorporating clinical outcomes and possibly gait analysis are warranted to better clarify the functional implications of the observed radiographic changes.

Finally, our follow‐up was limited to 12 months, thus the possibility of a later normalisation of coronal ankle parameters in the MA group cannot be completely excluded. Nonetheless, Cho et al. [2] reported that hindfoot alignment improves within the first 6 weeks after TKA but then remains stable without further change at 2 years, suggesting a limited capacity for long‐term compensation.

## CONCLUSIONS

The coronal alignment strategy adopted during total knee arthroplasty influences the postoperative orientation of the ankle joint. Mechanical, but not kinematic, alignment is associated with significant changes in talar and tibial plafond inclination. These findings support the role of KA in minimising unintended alterations at the ankle level, thus preserving the patient's specific knee–ankle relationship.

## AUTHOR CONTRIBUTIONS

Stefano Campi and Edoardo Franceschetti were responsible for data collection and conceptualisation. Giancarlo Giurazza was responsible for writing of the manuscript and qualified as corresponding author. Andrea Tanzilli and Biagio Zampogna were responsible for data analysis. Pietro Gregori supervised data acquisition and analysis. Michele Paciotti was responsible for realisation of Figures and Tables. Marco Donantoni and Andrea Marinozzi were responsible for the radiographic analysis. Umile Giuseppe Longo and Rocco Papalia were responsible for reviewing and critically revise the manuscript. All authors have given final approval of the version to be published.

## CONFLICT OF INTEREST STATEMENT

The authors declare no conflicts of interest.

## ETHICS STATEMENT

The study was performed in accordance with the ethical standards as laid down in the 1964 Declaration of Helsinki and its later amendments. Institutional review board approval was obtained for this research (IRB n° 32.19 OSS). All patients provided legitimate informed consent.

## Data Availability

The data that support the findings of this study are available from the corresponding author, [G.G.], upon reasonable request.

## References

[1] Ali A , Sundberg M , Robertsson O , Dahlberg LE , Thorstensson CA , Redlund‐Johnell I , et al. Dissatisfied patients after total knee arthroplasty: a registry study involving 114 patients with 8‐13 years of followup. Acta Orthop. 2014;85(3):229–233. 24786904 10.3109/17453674.2014.916487 PMC4062787

[2] Campi S , Giurazza G , Franceschetti E , Tanzilli A , Gregori P , Hirschmann MT , et al. Femoral cartilage variability affects the accuracy of kinematic alignment and imageless navigation in total knee arthroplasty: a prospective study from the FP‐UCBM Knee Study Group. Knee Surg Sports Traumatol Arthrosc. 2025;33(10):ksa.12725. 10.1002/ksa.12725 40517419

[3] Chang CB , Jeong JH , Chang MJ , Yoon C , Song MK , Kang S‐B . Concomitant ankle osteoarthritis is related to increased ankle pain and a worse clinical outcome following total knee arthroplasty. J Bone Jt Surg. 2018;100(9):735–741. 10.2106/JBJS.17.00883 29715221

[4] Choudhury AK , Bansal S , Pranav J , Raja BS , Gupta T , Paul S , et al. Increased medial talar tilt may incite ankle pain and predispose ankle osteoarthritis after correction of severity of knee varus deformity among patients undergoing bilateral total knee arthroplasty: a prospective observation. Knee Surg Relat Res. 2024;36(1):7. 38268011 10.1186/s43019-024-00212-x PMC10807238

[5] Franceschetti E , Campi S , Gregori P , Giurazza G , Samuelsson K , Hirschmann MT , et al. No differences in terms of complications, readmissions, reoperations, and patient‐reported outcomes in simultaneous bilateral versus staged bilateral total knee arthroplasty in selected patients. Knee. 2024;47:151–159. 38394994 10.1016/j.knee.2023.11.013

[6] Franceschetti E , Giurazza G , Campi S , Howell SM , Nedopil AJ , Papalia GF , et al. The “over‐the‐top” technique allows for accurate and reproducible restoration of the native tibial slope in kinematically aligned total knee arthroplasty: a retrospective comparative analysis from the FP‐UCBM Knee Study Group. Knee Surg Sports Traumatol Arthrosc. 2025;1:ksa.12750. 10.1002/ksa.12750 PMC12684349 40622130

[7] Gao F , Ma J , Sun W , Guo W , Li Z , Wang W . Radiographic assessment of knee‐ankle alignment after total knee arthroplasty for varus and valgus knee osteoarthritis. Knee. 2017;24(1):107–115. 27856127 10.1016/j.knee.2016.09.023

[8] Giurazza G , Campi S , Hirschmann MT , Franceschetti E , Tanzilli A , Gregori P , et al. Cartilage thickness can be accurately measured intraoperatively in total knee arthroplasty: a step further in calipered kinematic alignment. J Exp Orthop. 2025;12(1):e70155. 10.1002/jeo2.70155 39867675 PMC11763056

[9] Giurazza G , Caria C , Campi S , Franceschetti E , Papalia GF , Basciani S , et al. Femoral cartilage thickness measured on MRI varies among individuals: time to deepen one of the principles of kinematic alignment in total knee arthroplasty. A systematic review. Knee Surg Sports Traumatol Arthrosc. 2025;33(2):634–645. 39135541 10.1002/ksa.12408

[10] Giurazza G , Perricone G , Franceschetti E , Campi S , Gregori P , Zampogna B , et al. Femorotibial angle on short knee radiographs fails to accurately predict the lower limb mechanical alignment. A systematic review and meta‐analysis on different femorotibial angle definitions and short knee radiograph types. Orthop Rev. 2024;16:120053. 10.52965/001c.120053 PMC11213696 38947178

[11] Graef F , Falk R , Tsitsilonis S , Perka C , Zahn RK , Hommel H . Correction of excessive intraarticular varus deformities in total knee arthroplasty is associated with deteriorated postoperative ankle function. Knee Surg Sports Traumatol Arthrosc. 2020;28(12):3758–3765. 31776626 10.1007/s00167-019-05812-9

[12] Gunaratne R , Pratt DN , Banda J , Fick DP , Khan RJK , Robertson BW . Patient dissatisfaction following total knee arthroplasty: a systematic review of the literature. J Arthroplasty. 2017;32(12):3854–3860. 28844632 10.1016/j.arth.2017.07.021

[13] Gursu S , Sofu H , Verdonk P , Sahin V . Effects of total knee arthroplasty on ankle alignment in patients with varus gonarthrosis: Do we sacrifice ankle to the knee? Knee Surg Sports Traumatol Arthrosc. 2016;24(8):2470–2475. 26590564 10.1007/s00167-015-3883-2

[14] Howell SM , Kuznik K , Hull ML , Siston RA . Results of an initial experience with custom‐fit positioning total knee arthroplasty in a series of 48 patients. Orthopedics. 2008;31(9):857–863. 18814593 10.3928/01477447-20080901-15

[15] Jin G , Fan Y , Jiang L , Chen Z , Wang C . MAKO robot‐assisted total knee arthroplasty cannot reduce the aggravation of ankle varus incongruence after genu varus correction ≥ 10°: a radiographic assessment. BMC Musculoskelet Disord. 2023;24(1):492. 37322501 10.1186/s12891-023-06597-2 PMC10268520

[16] Kikuchi N , Kanamori A , Kadone H , Okuno K , Hyodo K , Yamazaki M . Varus knee osteoarthritis with ankle osteoarthritis demonstrates greater hindfoot inversion and larger ankle inversion loading during gait following total knee arthroplasty compared to varus knee osteoarthritis alone. Knee Surg Sports Traumatol Arthrosc. 2024;32(9):2309–2317. 38738824 10.1002/ksa.12249

[17] Kim CW , Gwak HC , Kim JH , Lee CR , Kim JG , Oh M , et al. Radiologic factors affecting ankle pain before and after total knee arthroplasty for the varus osteoarthritic knee. J Foot Ankle Surg. 2018;57(5):865–869. 29779992 10.1053/j.jfas.2018.02.002

[18] Kim JT , Han J , Lim S , Shen QH , Won YY . Kinematically aligned TKA aligns the ankle joint line closer to those of the native ankle than mechanically aligned TKA in bipedal stance. J Knee Surg. 2019;32(10):1033–1038. 31434142 10.1055/s-0039-1694796

[19] Lee JH , Jeong BO . Radiologic changes of ankle joint after total knee arthroplasty. Foot Ankle Int. 2012;33(12):1087–1092. 23199858 10.3113/FAI.2012.1087

[20] Nazlıgül AS , Doğan M , Duran İ , Moya‐Angeler J , Akkaya M . Mid‐term clinical and radiological changes in the ankle joint in varus knee osteoarthritis following total knee arthroplasty. J Clin Med. 2024;13(16):4700. 39200842 10.3390/jcm13164700 PMC11354923

[21] Onoi Y , Kamenaga T , Nakano N , Tsubosaka M , Kuroda Y , Hayashi S , et al. Achieving a neutral hip‐to‐calcaneus axis in kinematically aligned total knee arthroplasty equalizes coronal hindfoot pressure balance at initial ground contact in the gait cycle. Bone Joint J. 2025;107–B(6):604–614. 10.1302/0301-620X.107B6.BJJ-2024-1191.R1 40449893

[22] Rivière C , Iranpour F , Auvinet E , Howell S , Vendittoli P‐A , Cobb J , et al. Alignment options for total knee arthroplasty: a systematic review. Orthop Traumatol Surg Res. 2017;103(7):1047–1056. 28864235 10.1016/j.otsr.2017.07.010

[23] Rühling M , Kirschbaum SM , Perka C , Graef F . Increased ankle pain after total knee arthroplasty is associated with a preoperative lateralized gait and talar tilt, but not with ankle laxity or the range of motion of the subtalar joint. Bone Joint J. 2023;105–B(11):1159–1167. 10.1302/0301-620X.105B11.BJJ-2023-0419.R1 37907076

[24] Sangaletti R , Montagna A , Calandra G , Andriollo L , Bna C , Benazzo F , et al. Robotic functional alignment in knee arthroplasty minimizes impact on ankle alignment: role of MPTA and LDFA preservation. Knee Surg Sports Traumatol Arthrosc. 2025;33(6):2222–2229. 39905723 10.1002/ksa.12615

[25] Shichman I , Ben‐Ari E , Sissman E , Oakley C , Schwarzkopf R . Effect of total knee arthroplasty on coronal alignment of the ankle joint. J Arthroplasty. 2022;37(5):869–873. 35093550 10.1016/j.arth.2022.01.059

[26] Son HS , Choi JG , Ahn J , Jeong BO . Hindfoot alignment change after total ankle arthroplasty for varus osteoarthritis. Foot Ankle Int. 2021;42(4):431–439. 33218258 10.1177/1071100720970937

[27] Wang B , Saltzman CL , Chalayon O , Barg A . Does the subtalar joint compensate for ankle malalignment in end‐stage ankle arthritis? Clin Orthop Relate Res. 2015;473(1):318–325. 10.1007/s11999-014-3960-8 PMC4390960 25315275

